# Transcriptome analysis reveals a new virulence-associated trimeric autotransporter responsible for *Glaesserella parasuis* autoagglutination

**DOI:** 10.1186/s13567-024-01387-7

**Published:** 2024-10-07

**Authors:** Junxing Li, Shiyi Ye, Fei Su, Bin Yu, Lihua Xu, Hongchao Sun, Xiufang Yuan

**Affiliations:** Institute of Animal Husbandry and Veterinary Medicine, Zhejiang Academy of Agricultural Sciences, Hangzhou, 310021 China

**Keywords:** *Glaesserella parasuis*, transcriptome analysis, autoagglutination, *Wza*, *vtaA31*

## Abstract

Capsular polysaccharide is an important virulence factor of *Glaesserella parasuis*. An acapsular mutant displays multiple phenotype variations, while the underlying mechanism for these variations is unknown. In this study, we created an acapsular mutant by deleting the *wza* gene in the capsule locus. We then used transcriptome analysis to compare the gene expression profiles of the *wza* deletion mutant with those of the parental strain to understand the possible reasons for the phenotypic differences. The mutant Δ*wza*, which has a deleted *wza* gene, secreted less polysaccharide and lost its capsule structure. The Δ*wza* exhibited increased autoagglutination, biofilm formation and adherence to eukaryotic cells, while the complementary strain C-Δ*wza* partially restored the phenotype. Transcriptome analysis revealed several differentially expressed genes (DEGs) in Δ*wza*, including up-regulated outer membrane proteins and proteins involved in peptidoglycan biosynthesis, suggesting that *wza* deletion affects the cell wall homeostasis of *G. parasuis*. Transcriptome analysis revealed the contribution of non-coding RNAs in the regulation of DEGs. Moreover, a new virulence-associated trimeric autotransporter, VtaA31 is upregulated in Δ*wza*. It is responsible for enhanced autoagglutination but not for enhanced biofilm formation and adherence to eukaryotic cells in Δ*wza*. In conclusion, these data indicate that *wza* affects the expression of multiple genes, especially those related to cell wall synthesis. Furthermore, they provide evidence that *vtaA31* is involved in the autoagglutination of *G. parasuis*.

**Supplementary Information:**

The online version contains supplementary material available at 10.1186/s13567-024-01387-7.

## Introduction

*Glaesserella parasuis* (*G. parasuis*), previously known as *Haemophilus parasuis* (*H. parasuis*), is responsible for Glässer’s disease, which is characterised by polyserositis, polyarthritis, and meningitis. The bacteria involved are commensal organisms which colonise the upper respiratory tract of piglets as early as 2 days after birth. Under certain circumstances, they can also cause systemic infections [1, 2]. The disease is commonly found in domestic pig herds worldwide, and it is one of the leading causes of economic loss in the pig industry. The pathogenesis of *G. parasuis* is yet to be determined, although an increasing number of virulence factors have been identified [3, 4].

A total of 15 serovars have been identified. Although serovar was considered to be associated with virulence, the virulence of strains was not necessarily correlated to serovar [2, 5]. Serovars 4, 5 and 12 were the most common in pigs affected by Glässer’s disease. Strains in serovar 13 exhibited a higher likelihood of causing systemic infection [6–8]. Capsular polysaccharide serves as the determining antigen for the serovar, and the gene content involved in the synthesis of capsular polysaccharide has been identified in all 15 serovars [9, 10]. The deletion of genes in the capsule locus leads to increased biofilm production, heightened sensitivity to complement killing and phagocytosis by porcine alveolar macrophages, and the acapsular mutant displays reduced virulence to pigs [11, 12].

Increasing evidence shows that the expression of virulence factors in bacterial pathogens is coordinately regulated during different stages of infection to cope with the changing micro-environments in the host [13]. The expression of capsular polysaccharides and adhesins is coordinately regulated in several bacterial pathogens [14, 15]. The polysaccharide export protein (Wza) secretes polysaccharide to the surface of the bacteria to form the capsule structure, and this means that the *wza* deletion mutant causes the capsule to lose structure in several bacterial pathogens [16–19]. To study the impact of capsule deficiency on the expression of other virulence factors in *G. parasuis*, we created a *wza* deletion mutant from a serovar 13 isolate and then conducted a transcriptome analysis to examine the variation in gene expression variation in the mutant.

## Materials and methods

### Bacterial strains and growth conditions

The *Escherichia coli* (*E. coli*) and *G. parasuis* strains involved in this study are listed in Table 1. The *E. coli* was cultured with liquid LB medium (10 g tryptone, 10 g NaCl, and 5 g yeast extract per litre) or solid LB medium, supplemented with 1.5% agar. The *G. parasuis* strains were grown in a liquid TSB medium (35 g tryptone soya broth, 5 g yeast extract, 4 g proteose peptone, and 4 g tryptone per litre) or on a Tryptic Soy Agar (TSA) medium. Both of these were supplemented with 3% Foetal Bovine Serum (FBS) and 0.025% nicotinamide adenine dinucleotide (NAD). All the bacteria were cultured at 37 ℃, and a final concentration of kanamycin or gentamycin at 50 µg/mL for *E. coli* and 30 µg/mL for *G. parasuis* was supplemented when needed.

**Table 1:** **Strains and plasmid list**.

Strains	Description	Source	
*Escherichia coli*			
DH5a	F−, ϕ 80dlacZ ΔM15, Δ(lacZYA − argF )U169, deoR, recA1, endA1, hsdR17 (rK−, mK+), phoA, supE44, λ−, thi − 1, gyrA96, relA1	TaKaRa	
*Glaesserella parasuis*			
ZJ1208	Serovar 13 clinical isolate	Lab collection	
Δ*wza*	*wza* deletion mutant of ZJ1208, Kan^R^	This study	
C-Δ*wza*	*wza* complemented strain of Δ*wza*, Gen^R^	This study	
Δ*wza-vtaA*	*wza*, *vtaA31* double deletion mutant of ZJ1208, Kan^R^, Gen^R^	This study	
Plasmid			
pET 28a	Kan^R^, Template for PCR amplicon of kanamycin resistance gene	Laboratory collection	
pHERD	Gen^R^, Template for PCR amplicon of gentamycin resistance gene	Laboratory collection	
pUC 18	Amp^R^, using for construction of recombinant suicide vector	TaKaRa	
pUC-W	Amp^R^, suicide vector using for construction of *wza* deletion mutant	This study	
pUC-C	Amp^R^, suicide vector using for construction of *wza* complemented strain	This study	
pUC-V	Amp^R^, suicide vector using for construction of *wza*/*vtaA31* double deletion mutant	This study	

Kan: kanamycin; Gen: gentamycin; R: resistance.

### Strain construction

**Figure 1:**
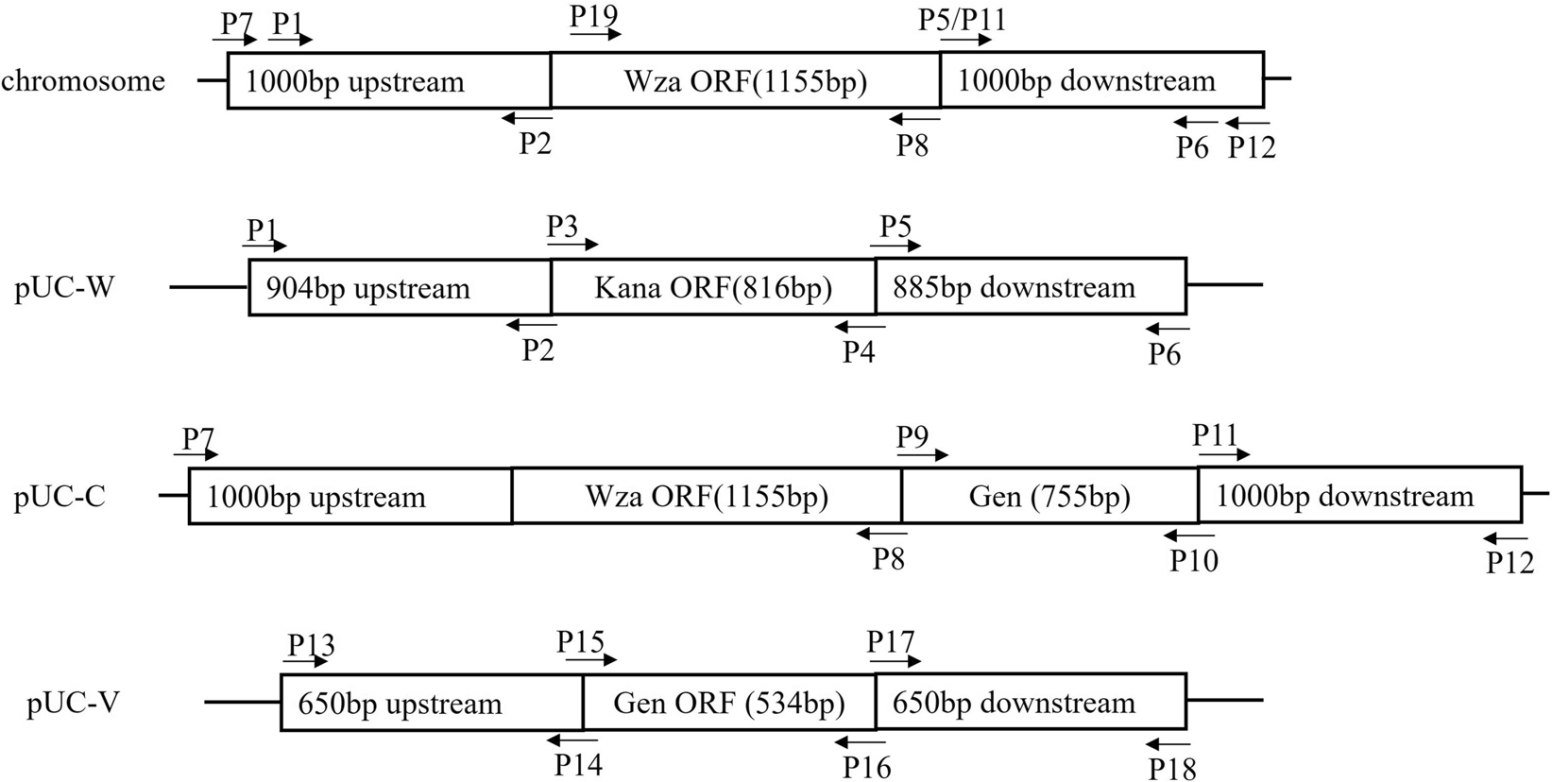
**Schematic diagrams of the target region of the** ***G. parasuis*** **chromosome and of the recombinant plasmids**.

The primers used for the construction of gene deletion and complementation mutants in this study are listed in Additional file 1. *Glaesserella parasuis* gene deletion mutants and complement strains were constructed and confirmed as described previously [20]. Homologous arms, target genes, and kanamycin or gentamycin-resistant gene amplicons were ligated through overlap PCR, as indicated in Figure 1, and cloned into pUC18 through restriction endonucleases. The resultant suicide plasmids for the deletion of *wza* and *vtaA31* were named pUC-W and pUC-V, respectively, and the recombinant plasmid for complementation of *wza* was named pUC-C. The suicide vector pUC-W was transformed into M-IV starvation medium-induced ZJ1208 competent cells and screened by kanamycin resistance for the *wza* deletion mutant designated as Δ*wza*. The *wza* complement strain and *wza*/*vtaA31* double deletion strain were obtained by transforming Δ*wza* competent cells induced in a M-IV starvation medium with pUC-C and pUC-V. The strains were then screened for gentamycin resistance, and the resultant strains were designated as C-Δ*wza* and Δ*wza-vta*, respectively. All the strains constructed in this study were identified by PCR to confirm the insertion or deletion of both the target genes and the gene of the selection markers (Additional file 2).

### Growth curve and colony morphology

To examine the impact of *wza* on growth, *G. parasuis* strains in liquid culture with a starting OD_600_ value of 0.6 were added to TSB (0.025% NAD, 3% FBS) at 1:100 dilution. The OD_600_ value of the samples was observed at 1 h intervals. Colonies on TSA plates were observed by optical microscope (BX51TRF, Olympus).

### Electron microscope

Samples for scanning electron microscope (SEM) were prepared using the previously reported method [21] and then analysed with SEM (SU8010, HITACHI).

Samples for transmission electron microscope (TEM) were fixed and labelled with polycationic ferritin as described previously [22], and were analysed with TEM (H-7650, HITACHI).

Samples for negative staining were conducted as follows. Bacteria cells suspended in water were dropped on polyvinyl formal-carbon-coated grids and absorbed for 2 min. The remaining water was removed with filter paper, and the bacteria attached to the grid were stained by 2% solution of phosphotungstic acid for 10 s. The remaining stain was removed with filter paper, and air-dried samples were observed with TEM (H-7650, HITACHI).

### Polysaccharide preparation and electrophoresis analysis

*G. parasuis* strains from overnight-cultured TSA plates were added to 50 mL TSB (0.025% NAD, 3% FBS) in a 1 L flask. The flask was incubated in a 37 ℃ shaker at 200 rpm until the OD_600_ value reached 1. The broth was centrifuged at 12 000 rpm for 15 min. The cell pellets and supernatant were then used to extract cell-associated capsular polysaccharides (CPS) and exopolysaccharides (EPS) secreted into the culture medium.

For CPS extraction, cell pellets were resuspended in 12 mL 0.1 M Tris (PH 8.5) supplemented with proteinase K by a final 200 µg/mL concentration and incubated at 56 ℃ for 1 h. Nucleic acids were removed by adding CaCl_2_ to 0.1 M and ethanol to 25%, as described previously [23]. To precipitate CPS, three times the volume of absolute ethanol was added and left at 4 ℃ overnight, followed by centrifugation at 8000 rpm for 20 min. The pelleted CPS was dissolved in 0.5 mL deionised water and incubated at 37 ℃ for 1 h, followed by centrifugation at 12 000 rpm for 5 min. The supernatant was stored at −20 ℃ before use.

For EPS extraction, nucleic acids were removed, and polysaccharide was purified using the same methods as for CPS. The EPS was dissolved in 20 mL of deionised water. Next, hexadecyl trimethyl ammonium bromide (CTAB) was slowly added to a concentration of 0.2%, and the mixture was held overnight at 4 ℃. After centrifugation for 20 min at 9000 rpm, the pellet was dissolved in 500 µL of 0.5 M NaCl solution. The solution was centrifuged at 12 000 rpm for 5 min, and the supernatant was mixed with four times the volume of absolute ethanol. The mixture was left at 4 ℃ overnight and centrifuged at 12 000 rpm for 15 min. Finally, the pellet was dissolved in 500 µL of deionized water and stored at −20 ℃ before use.

The polysaccharide samples were fractionated by SDS-PAGE and stained by Alcian blue, as described previously [24, 25].

### Autoagglutination assay

The autoagglutination assay was conducted as described previously but with some modifications [26]. The strains grown on TSA plates were added to TSB (0.025% NAD, 3% FBS) and cultured at 37 ℃, 200 rpm. The 15 mL aliquots of overnight liquid culture of each strain were added to 50-mL polypropylene tubes, which remained stationary for 12 h at room temperature. Samples were taken from the surface of the liquid culture at one-hour intervals, and the OD_600_ value of the samples was measured with a microplate reader (SpectraMax M5, Molecular Devices).

### Biofilm assay

Biofilm formation assay was conducted in 96-well microtiter plates as described previously [27], with some modifications. *G. parasuis* strains were added to TSB (0.025% NAD, 3% FBS) and cultured for 8 h, and the liquid cultures were adjusted to OD_600_ of 0.5 with TSB. Bacteria suspensions were added to the microtiter plate (Costar^®^ 3599, Corning) for 100 µL per well and placed in a 37 ℃ incubator for 18–36 h. TSB broth without bacteria was used as a negative control. The broth in the plate was removed after incubation, followed by two gentle washes with PBS. 0.1% (w/v) crystal violet of 100 µL per well was added and kept for 2 min at room temperature. The plate was washed twice and dried at room temperature. Finally, crystal violet in each well was dissolved in 100 µL of 70% (v/v) ethanol, and the OD_590_ value was measured with a microplate reader (SpectraMax M5, Molecular Devices).

### Adhesion assays

The adhesion assay was performed as previously described [28], with some modifications. PK15 cells were cultured in Dulbecco’s Modified Eagle Media (DMEM), supplemented with 10% foetal bovine serum (FBS) and 1% penicillin-streptomycin solution at 37 °C in a humidified 5% CO_2_ incubator. PK15 cells were seeded in 24-well plates with DMEM and cultured until reaching confluence. *G. parasuis* cultures were centrifuged and then resuspended in DMEM without antibiotics at 10^7^ c.f.u. mL^−1^. Confluent PK15 monolayers in 24-well plates were washed with D-Hanks solution three times, and then 1 mL aliquots of the bacterial suspensions were added to each well. After 2 h incubation in the humidified 5% CO_2_ incubator, the plates were washed with PBS three times to remove unattached *G. parasuis*. Cells in each well was incubated with 200 µL 0.05% trypsin/0.03% EDTA at 37 ℃ for 10 min, and then 800 µL ice-cold deionised PBS was added to each well. Cells were harvested through repeated pipetting, and a 100 µL sample was spread on the TSA plate for a colony count after 10-fold serial dilution. The adhesion level was expressed as log_10_ of average number of c.f.u recovered from four wells.

### Transcriptome sequencing and analysis

*Glaesserella parasuis* strains were cultured in TSB medium supplemented with NAD and serum, as mentioned above, until the OD_600_ value reached 0.7. Upon harvest, cells were immediately frozen in liquid nitrogen using centrifugation. The transcriptome sequencing and analysis were conducted at BGI Genomics Co., Ltd. Two biological replicates were used for each strain. In short, total RNA was extracted, followed by the removal of rRNA using biotin-labelled specific probes. The RNA was then purified and fragmented. Then, the cDNA library was created using Illumina’s TruSeq Stranded Total RNA Library Prep Kit and sequenced on an Illumina HiSeq 2500 platform.

Clean reads were obtained by removing low-quality reads, reads with sequence adaptors, reads with more than 5% “N” bases, and residual rRNA reads. Clean reads were mapped to the *G. parasuis* reference genome using HISAT2 (v2.0.1-beta) software. The Bowtie2 (v2.2.5) program was used to align the quality filtered reads to the assembled transcriptome, and gene expression levels were estimated using RSEM (v1.2.12). BSRD (Bacterial Small regulatory RNA Database) and Rfam (An RNA family database) were used for non-coding RNA (ncRNA) annotation. RNAplex (v2.3.4) was used for target gene prediction of ncRNAs. The DESeq2 package was used to analyse differentially expressed genes (DEGs) and ncRNAs, and expression levels with abs log_2_ (FoldChange) ≥ 1 & Adjusted *P* value ≤ 0.05 were considered as statistically significant.

### Quantitative real-time PCR (qrt-PCR)

To validate the results of the transcriptome sequencing, six genes were chosen for further analysis by qRT-PCR (Additional file 3). The 16s rRNA gene was utilised as an internal standard, as described previously [29]. Total RNA was extracted from *G. parasuis* strains using RNAprep Pure Cell/Bacteria Kit (Tiangen, Beijing, China). PrimeScript TM RT reagent Kit with gDNA Eraser (Takara, Otsu, Japan) was used to reverse transcript the total RNA. TB Green^®^ Premix Ex Taq™ II (Tli RNaseH Plus) was used for qPCR reaction on the ABI 7500 Real-Time PCR System (Applied Biosystems, USA). The fold change of gene expression in Δwza to ZJ1208 was analysed using the delta-delta comparative threshold cycle (2^–ΔΔCt^) method, and the relative quantification of gene expression was presented as log_2_ (2^−ΔΔCt^). Each qPCR was performed in triplicate.

### Western blot

To confirm the upregulation of VtaA31 expression, an equal volume of bacterial culture (OD_600_ value of 0.8) was used to prepare whole-cell proteins. The protein samples were separated by 12% SDS-PAGE and transferred to a polyvinylidene difluoride membrane using a Bio-rad Trans-Blot SD system. The membrane was blocked with PBST containing 10% skimmed milk, overnight at 4 °C. The membrane was incubated in 5% skimmed milk containing 1:100 diluted mouse serum immunised with recombinant truncated VtaA31 (Amino acid 738–1014), and goat anti-mouse IgG-HRP (1:3000) was used as the secondary antibody. The membrane was developed with Pierce ECL Western Blotting Substrate (Thermo Fisher Scientific) and imaged with Bio-rad ChemiDoc XRS + System and Image Lab Software.

### Statistical analysis

The data was first checked for normal distribution using the Shapiro-Wilk test in Graph Pad Prism 9.0 (GraphPad Software Inc., USA). Then, the statistical analysis was performed using one-way or two-way ANOVA or with methods recommended by the software. The results were shown as the means ± standard deviation (SD). The significant difference was defined as *p < 0.05, **p < 0.01, ***p < 0.001, ****p < 0.0001, respectively.

## Results

### The *wza* gene deficiency decreases capsular polysaccharide production

**Figure 2:**
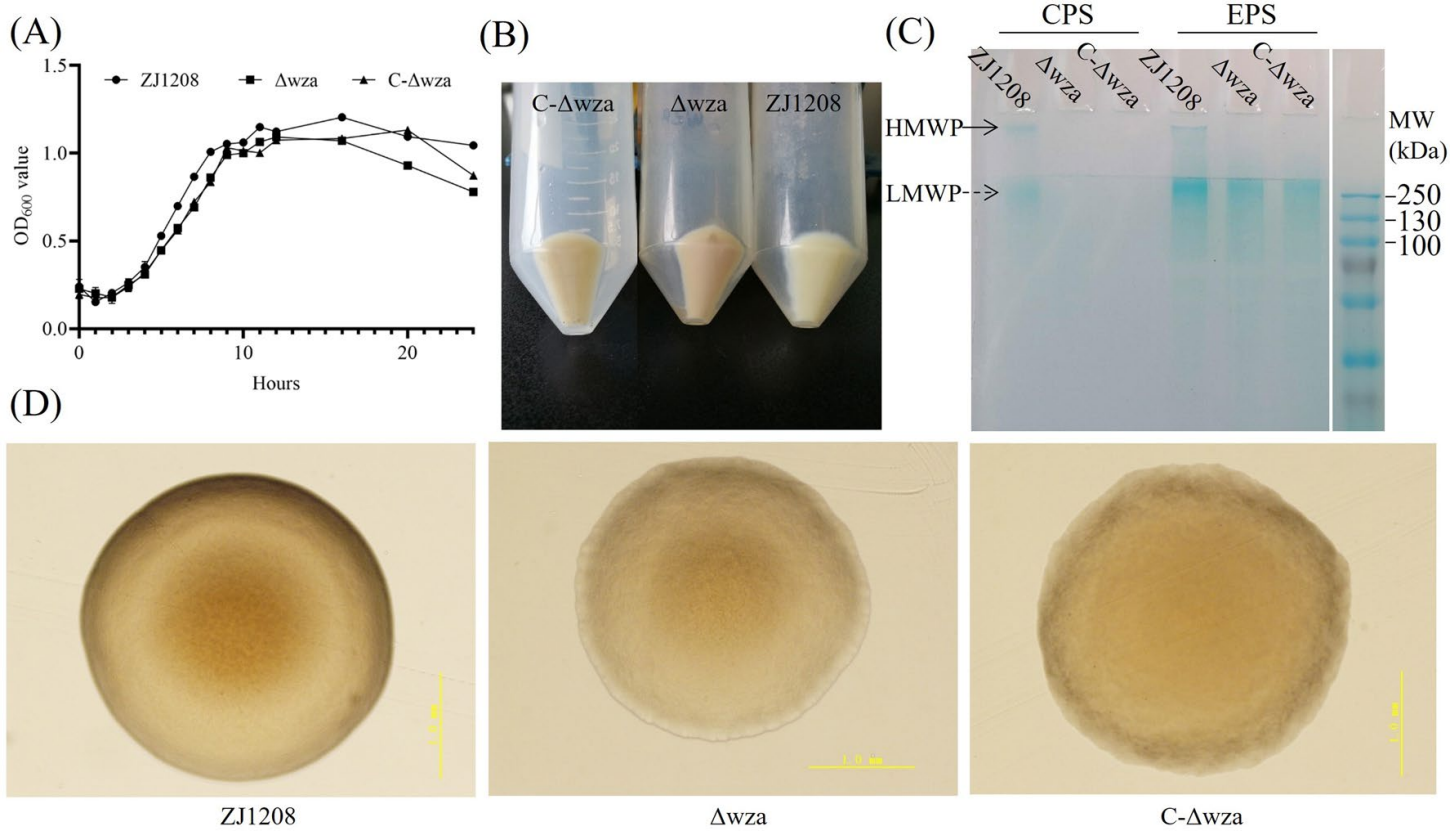
**Growth characteristic and capsular polysaccharide production**. **A** The wild strain ZJ1208, Δ*wza*, and C-Δ*wza* were cultured in TSB supplemented with 0.025% NAD and 3% bovine serum. The experiments were performed three times independently in triplicate. The means ± standard deviations from one representative experiment are shown. **B** A total of 50 mL of wild strain ZJ1208, Δ*wza*, and C-Δ*wza* were grown in TSB medium supplemented with 3% FBS and 0.025% nicotinamide adenine dinucleotide (NAD) in 500 mL flasks respectively until the OD_600_ value reached 0.8, and then the cultures were transferred to 50 mL tubes to centrifuge. **C** SDS-PAGE and alcian blue staining analysis of cell-associated capsular polysaccharide (CPS) and exopolysaccharide secreted to the culture medium (EPS). **D** The wild strain ZJ1208, Δ*wza*, and C-Δ*wza* were streak-inoculated on TSA plates for 48 h, and colonies were observed by optical microscope (Magnification, × 40).

The *wza* deletion mutant Δ*wza* showed a similar growth rate to the parent strain ZJ1208, which indicates that deleting the *wza* gene does not interfere with the growth of *G. parasuis* (Figure 2A). The precipitation colour of ZJ1208 is ivory, while the Δ*wza* appears brown; C-Δ*wza* partially restored the precipitation colour (Figure 2B). Two major bands of polysaccharides were observed in the extracts from the wild strain ZJ1208. The low molecular weight polysaccharide (LMWP) was distributed mainly at around 250 kDa, and the high molecular weight polysaccharide (HMWP) was too large to run from the stacking gel to the separating gel (Figure 2C). The EPS secreted into the culture medium displays a similar molecular weight profile as the cell-associated CPS, indicating that the LMWP and the HMWP are loosely associated with the cell. Deleting the *wza* gene aborts the production of cell-associated polysaccharides in terms of both LMWP and HMWP. At the same time, only the LMWP could still be observed in the culture medium extract. This suggests that *wza* gene deletion does not affect the secretion of LMWP, and the attachment of LMWP to the cell surface is HMWP dependent. Although the *wza* gene could be detected in C-Δwza (Additional file 2), the production of CPS could not be restored. The colony of ZJ1208 appears smooth, with even and protruding edges, but the Δ*wza* shows a flat colony with a slightly wrinkled surface and an irregularly undulated margin (Figure 2D). This indicates that capsulation is involved in determining the colony morphology of *G. parasuis*.

### The *wza *gene deficiency increases biofilm formation and PK15 cell adhesion

**Figure 3:**
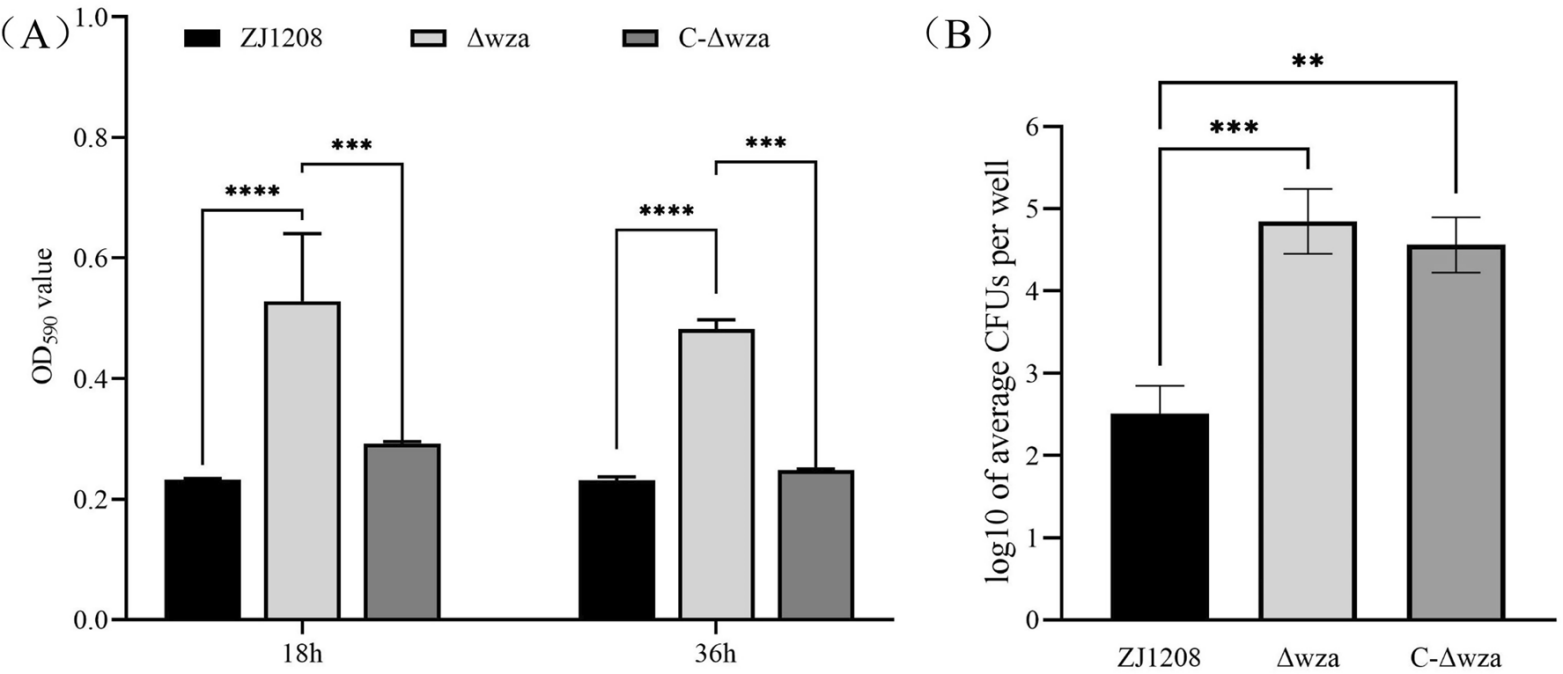
**Biofilm formation and PK15 cell adherence**. **A** Each bacteria suspension was added to eight duplicate wells of the microtiter plate and placed in a 37 ℃ incubator for 18–36 h. Biofilm formation was determined by staining with crystal violet and measuring the OD_590_ value after dissolving in ethanol. **B** Each bacteria suspension was added to four duplicate wells of a 24-well plate; the bacteria adherence to PK15 cells were collected and spread on TSA plates for CFU counting after proper dilution. The adhesion level was expressed as log_10_ of the average number of CFUs recovered from four wells. These experiments were performed independently three times, and the results were shown as means ± standard deviations (asterisks express statistical significance between the two strains: * *p* ≤ 0.05, ** *p* ≤ 0.01, *** *p* ≤ 0.001).

ZJ1208 formed a weak biofilm, and Δ*wza* formed significantly more biofilm than the wild strain. Robust biofilm formation of Δ*wza* occurred after 18 h of cultivation, and the biofilm did not further increase after another 36 h of cultivation (Figure 3A). C-Δ*wza* restored the weak biofilm formation phenotype, although the capsule was not restored. This indicates that it is not the capsule structure, but the expression of the *wza* gene, that affects the formation of biofilm. The adherence level of Δ*wza* increased significantly compared with the wild strain ZJ1208 (Figure 3B). C-Δ*wza* did not restore the adherence phenotype, although the adherence level showed a slight reduction, indicating that the capsule structure impedes adhesion. These results show that *wza* deficiency enhances bacterial adhesion to both abiotic and biotic surfaces.

### The *wza *gene deficiency causes growth phase-dependent autoagglutination

**Figure 4:**
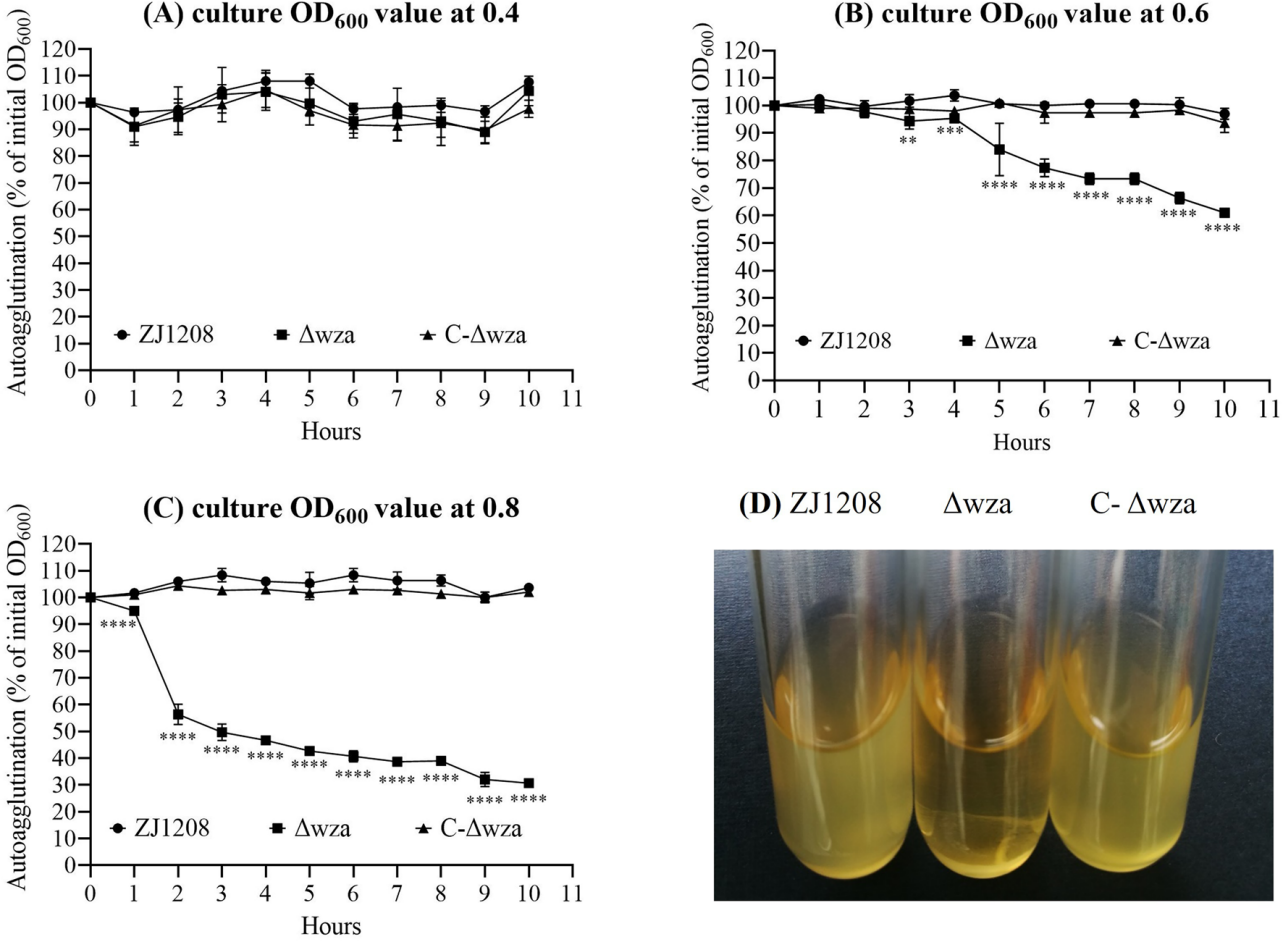
**Autoagglutination at different growth stages**. Liquid cultures of early-logarithmic (**A**), mid-logarithmic (**B**), and late-logarithmic (**C**) phases of growth in 50 mL tubes remained stationary for 12 h at room temperature. Samples were taken from the surface of the liquid culture at one-hour intervals, and the OD_600_ value of the samples was measured with a microplate reader. The experiment was performed three times, and the means ± standard deviations from three experiments are shown. **D** Liquid cultures of late-logarithmic phase remained stationary for 10 h (asterisks express statistical significance between ZJ1208 and Δ*wza*: * *p* ≤ 0.05, ** *p* ≤ 0.01, *** *p* ≤ 0.001, **** *p* ≤ 0.0001).

The wild strain ZJ1208 showed no autoagglutination during the 10-hour observation. The Δ*wza* did not show autoagglutination in the early-logarithmic phase, but exhibited obvious autoagglutination in the mid-logarithmic phase (Figures 4A and B). Autoagglutination increased significantly in the late-logarithmic growth phase, and the OD_600_ value declined to nearly 50% of the initial value after two hours of standing (Figure 4C). The phenotype of autoagglutination was fully restored in the *wza* gene complemented strain C-Δ*wza*. In addition, the liquid culture of C-Δ*wza* showed no autoagglutination after 10 h of standing (Figure 4D).

### No fimbria-like structures were observed on the surface of the cell

**Figure 5:**
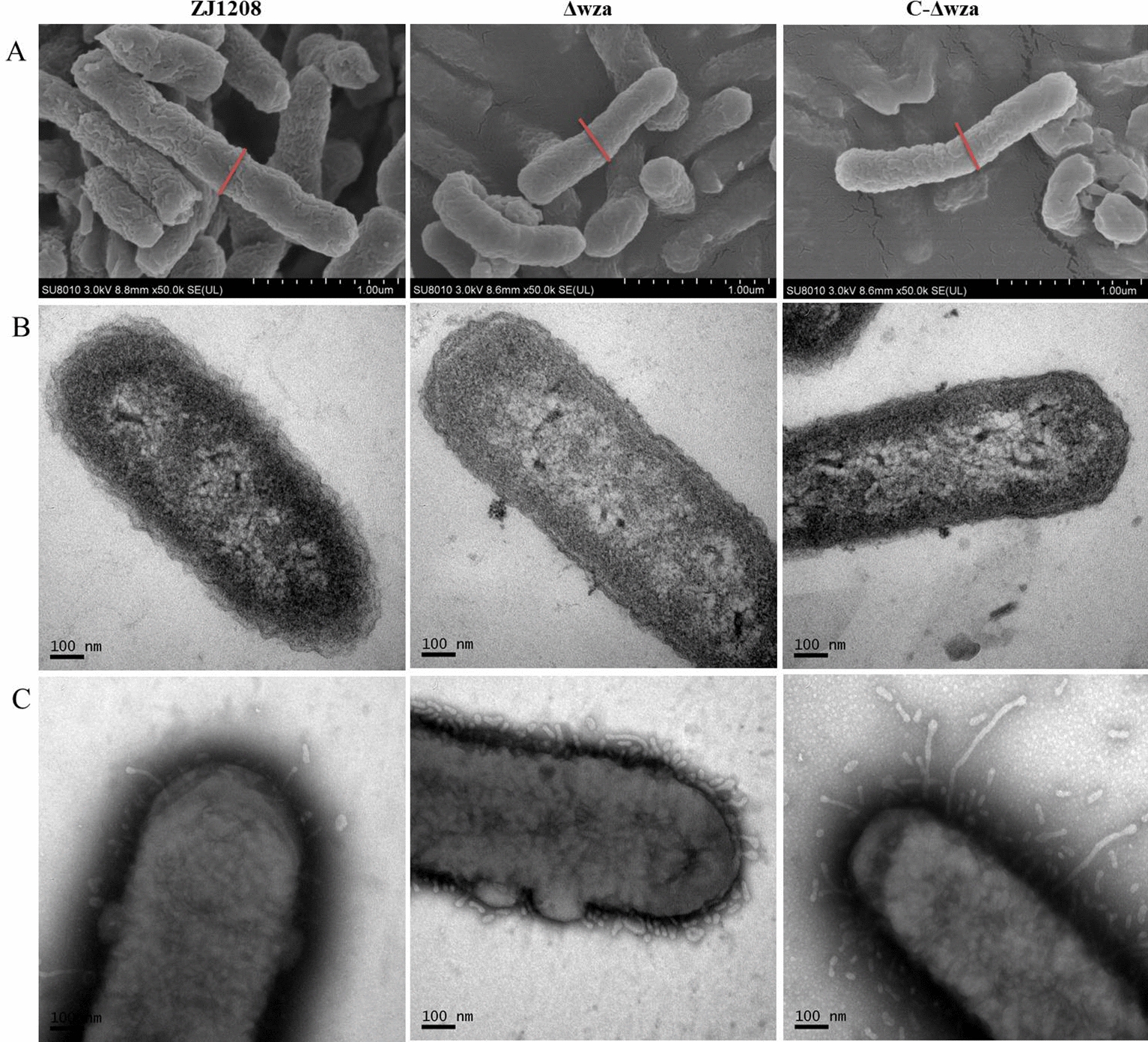
**Observation of ZJ1208**,** Δ*wza***, **and C-Δ*wza*by electron microscopes.** SEM (**A**), TEM (**B**) and negative staining (**C**) images of *G. parasuis* strains at late-logarithmic growth phase. The red bars indicate the same length of approximately 350 nm.

Fimbriae are involved in biofilm formation, cell adhesion, and autoagglutination of many bacterial pathogens. Hence, electron microscopes were used to check the presence of fimbriae structures. They were also used to observe capsule structure. When observed by SEM, ZJ1208 cells were thicker in size, and the mucoid cell surface was observed. Both Δ*wza* and C-Δ*wza* show less mucoid cell surface compared with ZJ1208 (Figure 5A). When observed by TEM, similar results were observed in terms of cell size and cell surface, which indicate that Δ*wza* lost capsule structure and C-Δ*wza* did not restore the capsule (Figure 5B). Negative staining revealed clear outer membrane vesicles (OMVs) around the cell surface of ZJ1208, and increased OMVs were observed for Δ*wza* and C-Δ*wza* (Figure 5C). No fimbria-like structures were observed by electron microscopes, which indicates that the phenotype change of Δ*wza* was not due to the fimbriae.

### Transcriptome analysis reveals multiple differentially expressed genes in Δ*wza*

**Figure 6:**
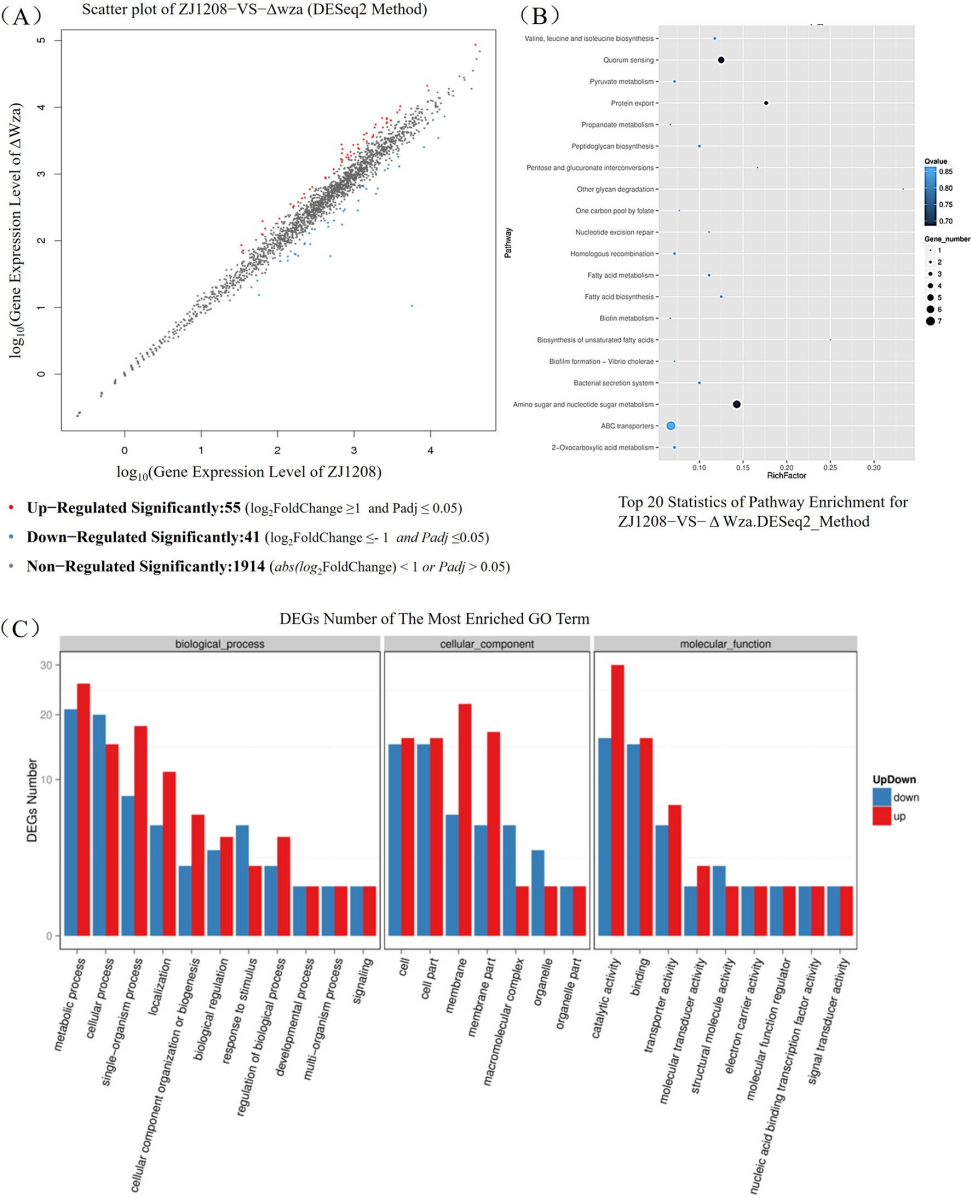
**Analysis of differentially expressed genes (DEGs) between ZJ1208 and Δ*wza***. **A** Comparison of gene expression between ZJ1208 and Δ*wza*. Each dot in the chart represents one annotated gene, and The DEGs are shown in red dots (significantly up-regulated) and blue dots (significantly down-regulated). Gene expression in Δ*wza* compared to that in ZJ1208, which meets abs log_2_(FoldChange) ≥ 1 & Adjusted *P* value ≤ 0.05 is considered significantly regulated. **B** Kyoto Encyclopaedia of Genes and Genomes (KEGG) pathway enrichment analysis of DEGs. The size of each dot shows the number of genes in the pathway. The colour of each dot indicates the q-value range. **C** Gene Ontology (GO) analysis of DEGs. DEGs numbers of both up-regulation (red bar) and down-regulation (blue bar) of the most enriched GO term are shown.

To investigate the genetic basis for the phenotypic variation in Δ*wza*, we compared the transcriptome data between Δ*wza* and ZJ1208. A total of 96 DEGs were identified in Δ*wza*. This consisted of 55 significantly up-regulated genes and 41 significantly down-regulated genes (Figure 6A). Details of all DEGs are listed in Additional file 4. According to the KEGG analysis, the most abundant pathways of DEGs include ABC transporters, amino sugar and nucleotide sugar metabolism, and quorum sensing (Figure 6B). This indicates that *wza* deficiency affects the transmembrane transport of substances, sugar metabolism, and communication with bacteria. DEGs of the most enriched gene ontology (GO) terms were classified into three categories: cellular components, biological processes, and molecular functions (Figure 6C). The most abundant GO terms in the three categories are metabolic processes, cells, and catalytic activities respectively.

Autoagglutination is typically facilitated by self-recognising surface structures, such as proteins and exopolysaccharides, and is also often one of the initial stages in biofilm formation [30]. Since the decrease in polysaccharide export and the absence of fimbria structures on the cell surface of Δ*wza*, we focused on the DEGs of outer membrane proteins (OMPs) for further analysis. Three proteins (HAPS_RS01105, HAPS_RS01760, and HAPS_RS11190) were classified as OMPs in the GO term of cellular component, all of which were up-regulated in Δ*wza* (Additional file 4). HAPS_RS01105 (outer membrane translocation and assembly module TamA) and HAPS_RS01760 (iron-regulated outer membrane protein CirA) have not been shown to be correlated with autoagglutination, and only CirA has been associated with biofilm and adhesion in *Salmonella* Enteritidis [31]. HAPS_RS11190 (a virulence-associated trimeric autotransporter designated as VtaA31 in this study) belongs to the family of trimeric autotransporter adhesins (TAAs) which plays prominent roles in autoagglutination, biofilm formation, adherence, and other pathogenic functions in many Gram-negative bacteria [32]. Hence, the up-regulation of *vtaA31* was possibly responsible for the phenotype variation in Δ*wza*. Moreover, TamB (HAPS_RS01110), partner protein of TamA, was also significantly up-regulated in Δ*wza*, and TamAB has shown to play a function in outer membrane homeostasis [33].

**Figure 7:**
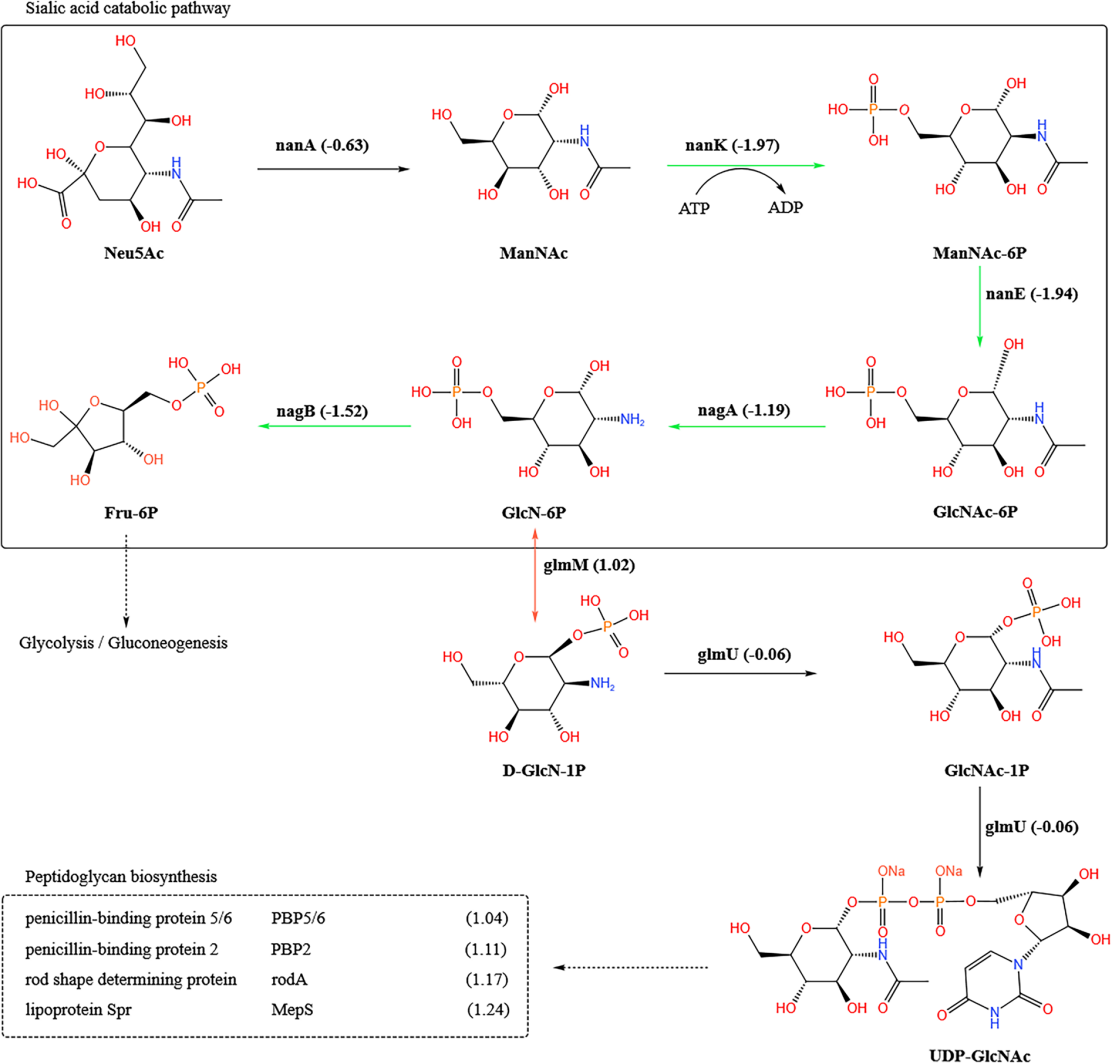
**Schematic representation of the sialic acid catabolic pathway in the Gram-negative bacteria and the comparative transcriptome analysis in ZJ1208 and its derived*wza*mutant**,** Δ*wza***. The metabolic pathway of sialic acid is shown in the solid line box, and the DEGs associated with peptidoglycan biosynthesis are displayed in the dashed line box. Enzymes used in each reaction are shown along with the solid arrow, and the number in each bracket indicates the fold of gene expression change represented as log_2_FoldChange (Δ*wza*/ZJ1208). Significantly up-regulated and down-regulated DEGs are highlighted as red and green solid arrows respectively. Dashed arrows indicate the downstream metabolism pathway. The chemical structures are drawn using KingDraw. NanA: N-acetylneuraminate lyase; NanK: N-acylmannosamine kinase; NanE: N-acylglucosamine-6-phosphate 2-epimerase; NagA: N-acetylglucosamine-6-phosphate deacetylase; NagB: glucosamine-6-phosphate deaminase; GlmM: phosphoglucosamine mutase. GlmU, a bifunctional UDP-N-acetylglucosamine pyrophosphorylase, has been shown to be bifunctional and also to possess the activity of UDP-N-acetylglucosamine diphosphorylase.

In the pathway of amino sugar and nucleotide sugar metabolism, we identified six DEGs closely associated with sialic acid metabolism. All five major enzymes directly involved in sialic acid metabolic were down-regulated in Δ*wza*, four of which were significantly down-regulated (Figure 7). GlmM was significantly up-regulated in Δ*wza*, which is required to produce glucosamine-1-phosphate (D-GlcN-1P), an early intermediate of peptidoglycan synthesis. Moreover, four proteins associated with peptidoglycan biosynthesis were significantly up-regulated in Δ*wza*, and no significantly down-regulated DEGs in the peptidoglycan biosynthesis pathway were detected (Figure 7, Additional file 4). These data indicate that sialic acid is more likely to be allocated to the peptidoglycan synthesis pathway rather than the glycolytic pathway in Δ*wza*, and that peptidoglycan synthesis is enhanced due to *wza* deficiency.

### Transcriptome analysis reveals contribution of ncrnas in the regulation of degs

**Figure 8:**
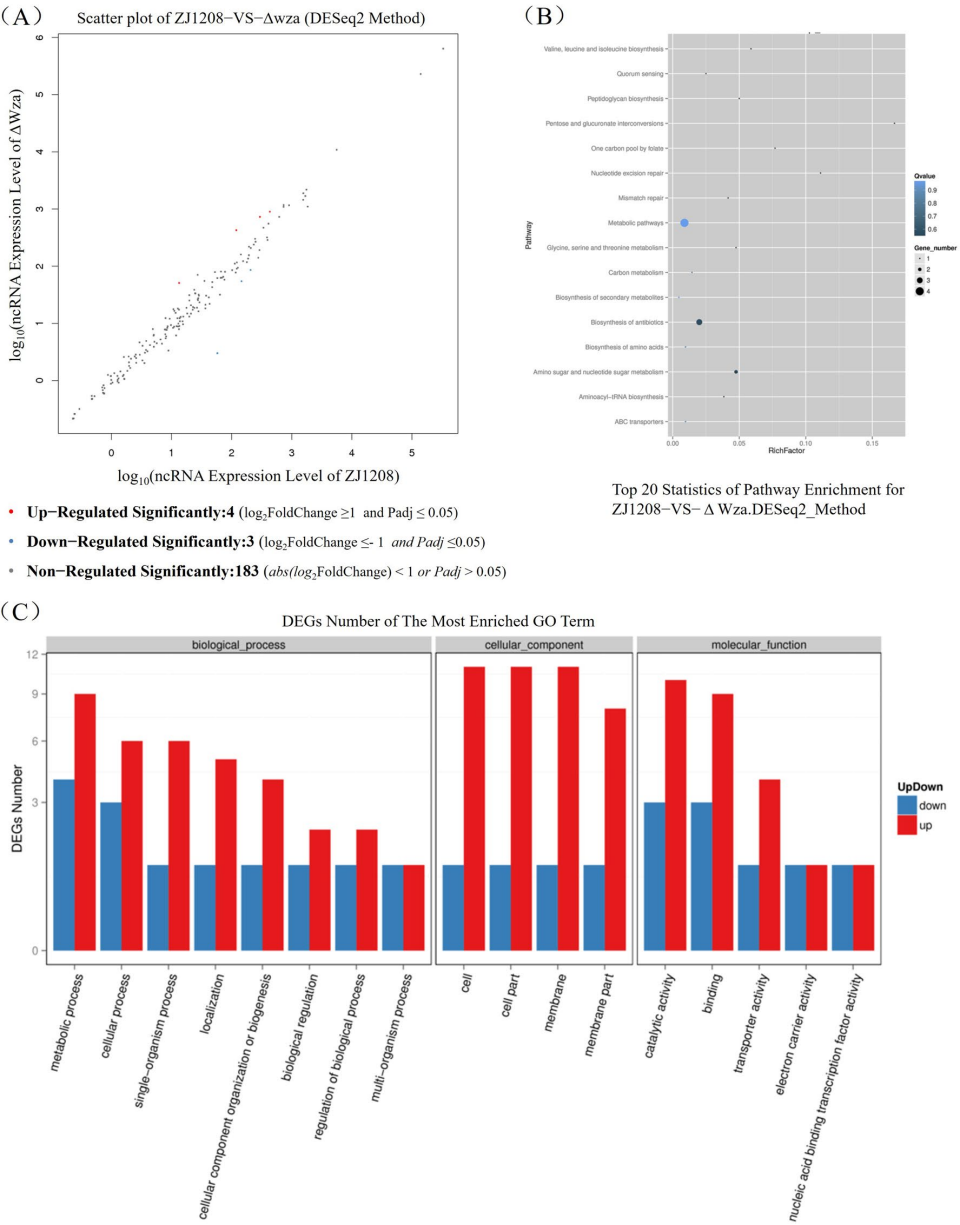
**Analysis of differentially expressed ncRNAs between ZJ1208 and Δ*wza***. **A** Comparison of ncRNA expression between ZJ1208 and Δ*wza*. Each dot in the chart represents one annotated ncRNA, and the differentially expressed ncRNAs are show in red dots (significantly up-regulated) and blue dots (significantly down-regulated). NcRNAs in Δ*wza* compared to that in ZJ1208 which meet abs log_2_(FoldChange) ≥ 1 & Adjusted *P* value ≤ 0.05 are considered significantly regulated. **B** Kyoto Encyclopedia of Genes and Genomes (KEGG) pathway enrichment analysis of DEGs targeted by differentially expressed ncRNAs. The size of each dot shows the number of genes in the pathway. The colour of each dot indicates the q-value range. **C** Gene Ontology (GO) analysis of DEGs targeted by differentially expressed ncRNAs. DEGs numbers of both up-regulation (red bar) and down-regulation (blue bar) of the most enriched GO term are shown.

To identify the differentially expressed ncRNAs (DEncRNAs) that responded to *wza* deficiency, we compared the expression of ncRNAs in Δ*wza* with that in ZJ1208. This comparison revealed seven DEncRNAs in Δ*wza*, including four significantly up-regulated ncRNAs and three significantly down-regulated ncRNAs (Figure 8A). Details of DEncRNAs and the predicted target genes can be found in Additional file 5 and Additional file 6, respectively. To investigate the potential function of the ncRNAs in Δ*wza*, the DEncRNAs targeting the DEGs were subjected to KEGG and GO enrichment analysis. The most enriched KEGG pathways and GO terms (Figure 8) are highly similar to those shown in Figure 6, suggesting that DEncRNAs play a widespread role in regulating the expression of DEGs. For example, two (*nagA* and *glmM* in Figure 7) out of the six DEGs in the pathway of amino sugar and nucleotide sugar metabolism are target genes of DEncRNAs (Additional file 6). Interestingly, HAPS_RS11190 (*vtaA31*) is the target gene for six of the seven DEncRNAs (Additional file 5), including BGI_novel_N180, BGI_novel_N224, BGI_novel_N228, BGI_novel_N233, BGI_novel_N265, and BGI_novel_N278, which suggests that these ncRNAs are probably involved in the regulation of *vtaA31*.

### Validation of degs by qrt-PCR

**Figure 9:**
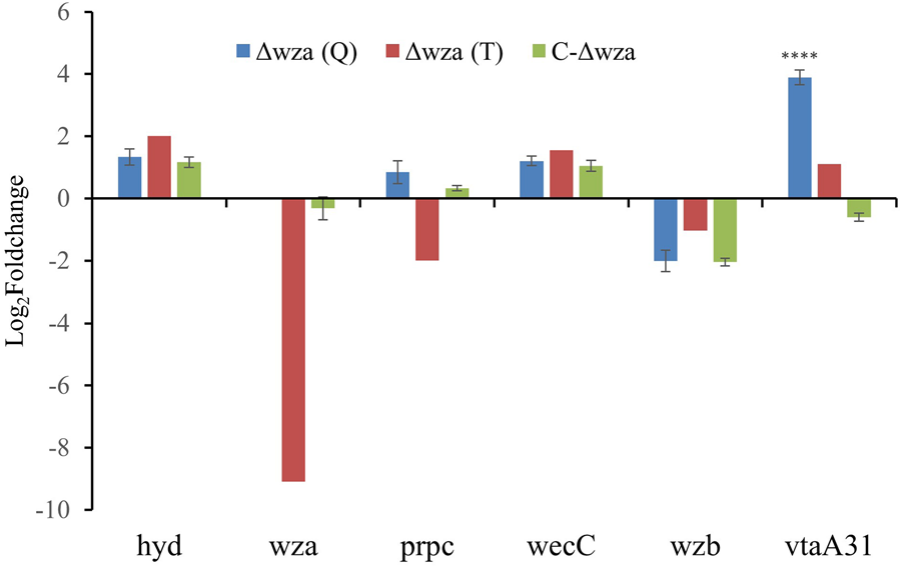
**Validation of DEGs by qRT-PCR**. Fold change of gene expression in Δ*wza* or C-Δ*wza* to ZJ1208 was analysed using the delta-delta comparative threshold cycle (2–ΔΔCt) method, and the relative quantification of gene expression was presented as log_2_ (2 − ΔΔCt). Δ*wza*(T) and Δ*wza*(Q) represent the results of transcriptome and qRT-PCR analysis, respectively. Statistical analysis was performed between Δ*wza*(Q) or C-Δ*wza* using two-way ANOVA. Data represent the mean ± SD of three independent experiments (asterisks express statistical significance between Δ*wza* and C-Δ*wza*: **** *p* ≤ 0.0001).

To confirm the transcriptome sequencing results, *wza*, *vtaA31*, and another four randomly selected DEGs were validated by qRT-PCR. The qRT-PCR results indicated that the expression levels of five out of the six DEGs were in line with those obtained through transcriptome sequencing. The only exception was the *prpc* gene, suggesting overall consistency between the results from qRT-PCR and transcriptome sequencing. No signal was detected for the *wza* gene in Δ*wza*, while an apparent signal close to ZJ1208 was detected in C-Δ*wza*, suggesting successful transcription of the *wza* gene in C-Δ*wza*. The transcription of *vtaA31* was significantly increased in Δ*wza* and restored in C-Δ*wza*, which indicates that the *wza* gene affects *vtaA31* expression (Figure 9).

### VtaA31 is involved in the autoagglutination of *G. parasuis*

**Figure 10:**
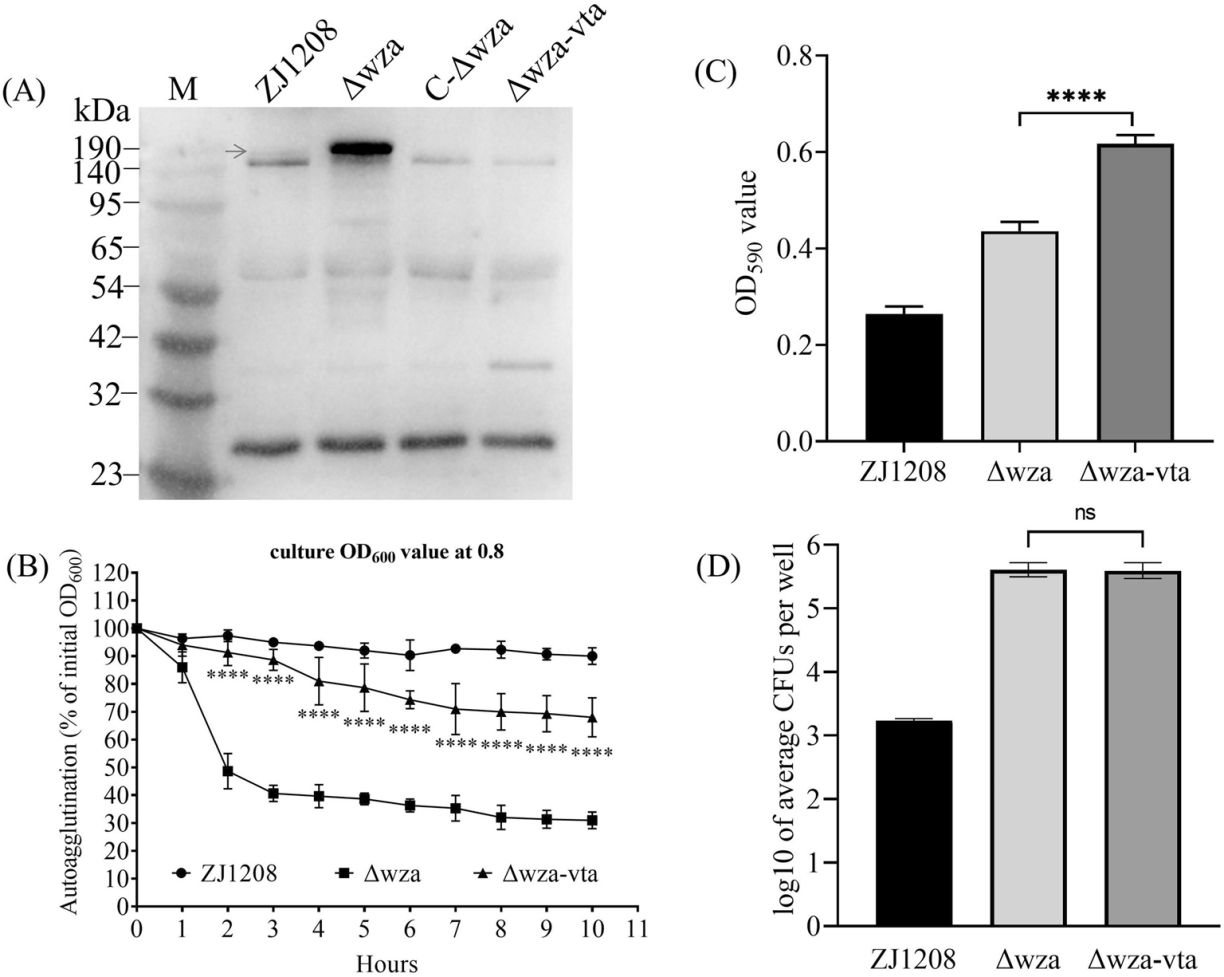
**Effect of*vtaA31*deletion on autoagglutination**,** biofilm formation and adherence of Δ*wza***. **A** Expression of VtaA31 in *G. parasuis* was identified by western blot. M: Protein marker and the red arrow indicate the 156.3 kDa VtaA31 protein. **B** Liquid culture of the late-logarithmic phase in 50 mL tubes remained stationary for 10 h at room temperature. Samples were taken from the surface of the liquid culture at one-hour intervals, and the OD_600_ value of the samples was measured with a microplate reader. **C** Each bacteria suspension was added to eight duplicate microtiter plate wells and placed in a 37 ℃ incubator for 24 h. Biofilm formation was determined by staining with crystal violet and measuring the OD590 value after dissolving in ethanol. **D** Each bacteria suspension was added to four duplicate wells of a 24-well plate. The bacteria adherence to PK15 cells was collected and spread on TSA plates for CFU counting after proper dilution. The adhesion level was expressed as log_10_ of the average number of CFUs recovered from four wells. The above experiments were performed three times, and the means ± standard deviations from three experiments are shown. Statistical analysis was performed between Δ*wza* or Δ*wza-vtaA* using one-way or two-way ANOVA (asterisks express statistical significance between Δ*wza* and Δ*wza-vtaA*: **** *p* ≤ 0.0001, ns: non-significant).

To determine the role of VtaA31 in the phenotype variation of Δ*wza*, the *vtaA31* gene (GenBank accession number PP540026) was deleted from Δ*wza* to create the double gene deletion mutant Δ*wza-vta*. VtaA31 was expressed at a very low level in ZJ1208. The expression level was significantly increased in Δ*wza*, and even lower expression levels were observed in C-Δ*wza* (Figure 10A). No VtaA31 was observed in Δ*wza-vta*, which confirms the deletion of *vtaA31* gene. It shows that the deletion of *vtaA31* significantly decreased the autoagglutination of Δ*wza*, indicating that *vtaA31* is involved in the autoagglutination of *G. parasuis* (Figure 10B). However, the autoagglutination of Δ*wza-vta* was faster than that of ZJ1208, suggesting the contribution of other factors to autoagglutination. Interestingly, the Δ*wza-vta* formed significantly more biofilm than Δ*wza*, which indicates that *vtaA31* affects biofilm formation (Figure 10C). Deletion of *vtaA31* had no effect on adherence to PK15 cells, suggesting a limited role of VtaA31 in *G. parasuis* adherence (Figure 10D).

## Discussion

*Glaesserella parasuis* is a significant bacterial pathogen of swine, often coinfected with other viral pathogens such as porcine reproductive and respiratory syndrome virus (PRRSV), porcine circovirus type 2 (PCV2), and influenza virus (IAV) [34, 35]. Due to a poor understanding of virulence factors, protective antigens, and a lack of cross-protection among different serovars, novel vaccine development for Glässer’s disease remains challenging. In this study, the *wza* gene of the capsular polysaccharide export protein was deleted in-frame and the impact of *wza* deficiency on overall gene expression in *G. parasuis* was investigated.

The virulence of *G. parasuis* strains varies widely, ranging from non-virulent to highly virulent. The ZJ1208 used in this study belonged to serovar 13, considered a virulent serovar. ZJ1208 was further confirmed to be virulent by two different “pathotyping” PCR assays based on a subset of virulence genes [36, 37]. The amplification results of all virulence genes were consistent in both ZJ1208 and mutants (Additional file 7).

The colony morphology of Δ*wza* was similar to ZJ1208 when observed with naked eyes, yet showed a clear difference when observed under microscopy. TEM observation and SDS-PAGE analysis of capsular polysaccharides confirm that Δ*wza* is acapsular. Interestingly, the lack of *wza* affects the association of both HMWP and LMWP to the cell surface and only affects the HMWP secretion to the medium. This suggests that LMWP may be exported by proteins other than Wza.

The cell precipitation colour of ZJ1208 cultured under sufficient oxygen appears ivory and turns brown when cultured under insufficient oxygen (data not shown). The cell precipitation colour of Δ*wza* cultured under sufficient oxygen is close to that of ZJ1208 cultured under inadequate oxygen. This similarity indicates that *wza* deficiency may affect the cellular responses of *G. parasuis* to oxygen availability. Meanwhile, anaerobic C4-dicarboxylate transporter DcuB (HAPS_RS10065) was significantly down-regulated in Δ*wza* in the transcriptome analysis in this study. Since DcuB is important for the growth of enteric bacteria under anaerobic conditions [38], the down-regulation of DcuB may impair Δ*wza*’s capacity to grow under oxygen limitation.

In this study, we observed enhanced biofilm formation in Δ*wza*, consistent with the capsular mutant of the serovar 5 strain HS069 [12]. An inverse correlation was observed between CPS production and biofilm formation in *Pasteurella multocida* Serogroup A [39]. Arginine transporter permease subunit artM was significantly up-regulated in the transcriptome analysis in this study, and it looked possible that artM may affect the biofilm formation of *H. parasuis* [40].

Enhanced adherence of Δ*wza* to the eukaryotic cell PK15 was observed in this study. The capsular mutant showed an equal level of cell adherence to a previous study [12]. The *capD* gene encoded a protein for polysaccharide biosynthesis protein. A *capD*-deficient mutant also exhibited increased adherence to eukaryotic cells [41]. The capsule structure concealed the Knh trimeric autotransporter adhesion of *Kingella kingae* and hindered the high-affinity adherence to human epithelial cells mediated by Knh in the absence of type IV pili retraction [42, 43]. In this study, no pili were observed on the surface of ZJ1208. It is thought that the enhanced adhesion of Δ*wza* to eukaryotic cells is due to the unmasking of adhesins such as VtaA. Although *vtaA31* was up-regulated in Δ*wza*, it is not the major factor responsible for the phenotype of enhanced adherence.

Enhanced autoagglutination was observed in several gene mutants of *G. parasuis* such as *clpP*, HAPS_0849, and *htrA* [26, 44, 45]. On the contrary, deletion of the *luxS* gene led to weakened autoagglutination of *H. parasuis* [46]. It seems that the phenotype of autoagglutination could be influenced by multiple genes, but the factors involved in autoagglutination have not been defined for *G. parasuis*. Since TAAs are common proteinaceous autoagglutinins for bacteria [30], the up-regulation of the TAA (HAPS_RS11190) in this study is considered to be the possible factor causing enhanced autoagglutination. Multiple *vtaAs* are present within the genome of one *G. parasuis* strain, and there is relatively high sequence homology among those *vtaAs* [47]. Four *vtaAs* from the ZJ1208, genome which shows the highest sequence homology with HAPS_RS11190, were selected to confirm changes in expression level by qRT-PCR. Only *vtaA31* showed a significantly up-regulated expression level (data not shown). The increased expression of VtaA31 was further confirmed by western blot, and the cross-reaction of the positive sera for VtaA31 with other potential VtaA proteins was observed, as previously reported [48]. Therefore, the *vtaA31* was in-frame deleted from Δ*wza* to check its functionality in autoagglutination. The reduced autoagglutination of the double gene deletion mutant Δ*wza-vta* confirmed that *vtaA31* is directly involved in autoagglutination. The majority of DEncRNAs were predicted to target the *vtaA31* gene, suggesting that *vtaA31* is likely regulated by ncRNAs. Further study is needed to understand the contributions of these DEncRNAs to the regulation of *vtaA31*.

The translocation and assembly module (TAM) is a nanomachine required for the virulence of bacterial pathogens. The TamAB system drives the assembly of proteins into bacterial outer membranes. It is believed that the TamAB system plays a role in outer membrane homeostasis [33, 49]. TamA and TamB were significantly up-regulated in Δ*wza*, and several OMPs were also significantly up-regulated in Δ*wza*, suggesting that the deletion of the *wza* gene may disrupt outer membrane homeostasis in *G. parasuis*.

Bacteria can incorporate many sugars, including glucose, and amino sugars such as N-acetylglucosamine. The allocation of sugar to metabolic pathways is tightly regulated. GlmS and NagB play a central role in determining whether sugars and amino sugars are directed into the glycolysis or cell wall biosynthesis pathways, and they regulate amino sugar metabolism in opposing directions [50–52]. The amino sugar, Neu5Ac, is the precursor of nearly all sialic acids. In this study, *nagB* and *glmS* were both found to be significantly regulated in Δ*wza*, while in opposite directions. The metabolic pathway of Neu5Ac is generally advantageous for cell wall synthesis in Δ*wza*, rather than for glycolysis. Hence, *wza* deficiency affects the distribution of sugar to the glycolysis or cell wall biosynthesis pathways in *G. parasuis*. Overall, our study elucidated the effect of *wza* deficiency on global gene expression and identified a virulence-associated trimeric autotransporter that is involved in the autoagglutination of *G. parasuis*.

## Supplementary Information

**Additional file 1: Primers for construction of gene deletion and complementation mutants**. Primer sequences and size of amplicons are listed.

**Additional file 2: PCR identification of gene deletion and complementary strain**. Lane M: DNA marker; lane 1: Amplicon of *wza* gene; lane 2: Amplicon of *vtaA31* gene; lane 3: Amplicon of kanamycin resistance gene; lane 4: Amplicon of gentamycin resistance gene.

**Additional file 3: Primers for Quantitative real-time PCR**. Target gene, primer sequences, amplicon size and source of primers are listed.

**Additional file 4: Differentially expressed genes between ZJ1208 and Δ*wza***. Information of differentially expressed genes are listed, including GeneID, length, log2FoldChange (Δ*wza*/ZJ1208) et al.

**Additional file 5: Differentially expressed ncRNAs between ZJ1208 and Δ*wza***. Information of differentially expressed ncRNAs are listed, including GeneID, length, expression level, log2FoldChange (Δ*wza*/ZJ1208) et al.

**Additional file 6: Predicted targets of all ncRNAs**. Information of predicted targets of all ncRNAs are listed.

**Additional file 7: Amplification results of two “pathotyping” PCR assays**. Information of amplification results of all virulence genes are listed.

## Data Availability

The nucleotide sequence data underlying this article is available in the GenBank Nucleotide Database under accession number PP540026. All other data generated or analysed during this study are included in this published article and its supplementary information files.
