# Supplementary material for: Transcriptome analysis reveals a new virulence-associated trimeric autotransporter responsible for Glaesserella parasuis autoagglutination
Source: Vet Res. 2024 Oct 7;55:130. doi: 10.1186/s13567-024-01387-7 (PMC11460128; doi:10.1186/s13567-024-01387-7)
Supplement: Supplementary file 1 — Additional file 1: Primers for construction of gene deletion and complementation mutants. Primer sequences and size of amplicons are listed. [file 13567_2024_1387_MOESM1_ESM.docx]

**Additional file 1 Primers for construction of gene deletion and complementation mutants**

| Primers (restriction endonuclease) | Sequence (5’→3’) | Amplicon (bp) |
| --- | --- | --- |
| P1 (EcoR I) | GCGGAATTCTGTGTTTGCAAGCATTGGCTATGGA | 923 |
| P2 | ATGGCTCATAATCCATCTTACTCATTTTAT |  |
| P3 | GTAAGATGGATTATGAGCCATATTCAACGGGAAAC | 839 |
| P4 | CCAAACATTAATTAGAAAAACTCATCGAGC |  |
| P5 | AGTTTTTCTAATTAATGTTTGGAAGTAT | 905 |
| P6 (Hind III) | GAGAAGCTTCTCTTTCATCTGAGGATTCTTAGGC |  |
| P7 (BamH I) | GAGGGATCCCGATTGAAGCTCAGGTCAAATCTTTTG | 2188 |
| P8 | GCTTATGTCAATTCGTTACCAATTCCTCACTCTTAA |  |
| P9 | AGAGTGAGGAATTGGTAACGAATTGACATAAGCCTGTTCG | 789 |
| P10 | TACTTCCAAACATTAATTAGGTGGCGGTACTTGGGTCG |  |
| P11 | AAGTACCGCCACCTGCTTATGTCAATTCGTTACCAATTCCTCACTCTTAAGAATTAATGTTTGGAAGTAT | 1034 |
| P12 (Hind III) | GAGAAGCTTTCGCAAACGAAAAATCTTCCGTT |  |
| P13 (BamH I) | CGTGGATCCACAGACAGGGATAAATATCGCTTGGGATGA | 680 |
| P14 | ATCGTTGCTGCTGCGTAACATAATAAGCTTCTCTACTGTTTTTCC |  |
| P15 | GGAAAAACAGTAGAGAAGCTTATTATGTTACGCAGCAGCAACGATGT | 578 |
| P16 | TTACTCAGATATTTAATATCTTAGGTGGCGGTACTTGGGTCGATA |  |
| P17 | ATATCGACCCAAGTACCGCCACCTAAGATATTAAATATCTGAGTA | 685 |
| P18 (Xho I) | GCGCTCGAGAAGCGTTTGCTCGTTGGTTAAACACT |  |
| P19 | GAGGGATCCACTGGTGTGATTTTATCTCT | 1080 (with P8) |
| P20 | GAGCCATGGGCATGAATAAAATATTTAGAGTTATTTGGAGTCATGCC | 4616 |
| P21 | GCCCTCGAGCCACTGTAATGCAATACCT |  |
