# Supplementary figures and images for: Transcriptome analysis reveals a new virulence-associated trimeric autotransporter responsible for Glaesserella parasuis autoagglutination
Source: Vet Res. 2024 Oct 7;55:130. doi: 10.1186/s13567-024-01387-7 (PMC11460128; doi:10.1186/s13567-024-01387-7)

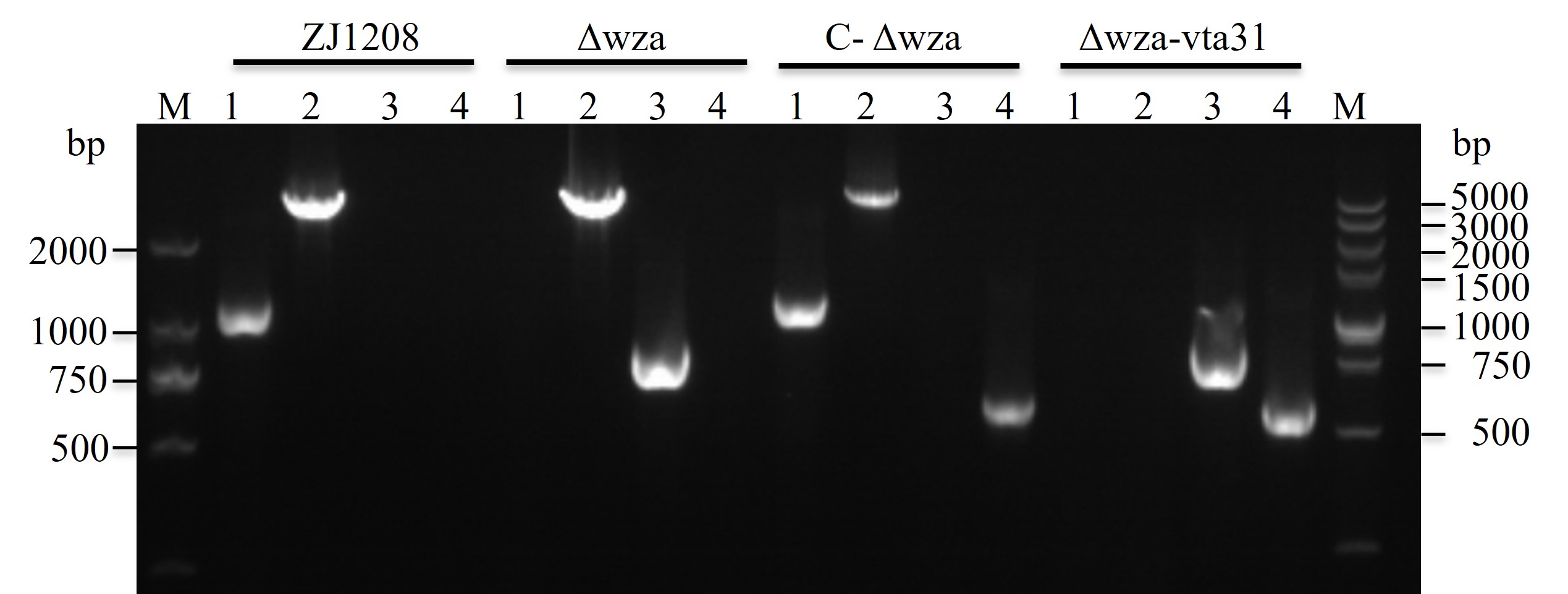

Supplement: Supplementary file 2 — Additional file 2: PCR identification of gene deletion and complementary strain. Lane M: DNA marker; lane 1: Amplicon of wza gene; lane 2: Amplicon of vtaA31 gene; lane 3: Amplicon of kanamycin resistance gene; lane 4: Amplicon of gentamycin resistance gene. [file 13567_2024_1387_MOESM2_ESM.jpg]
