# Supplementary material for: Transcriptome analysis reveals a new virulence-associated trimeric autotransporter responsible for Glaesserella parasuis autoagglutination
Source: Vet Res. 2024 Oct 7;55:130. doi: 10.1186/s13567-024-01387-7 (PMC11460128; doi:10.1186/s13567-024-01387-7)
Supplement: Supplementary file 3 — Additional file 3: Primers for Quantitative real-time PCR. Target gene, primer sequences, amplicon size and source of primers are listed. [file 13567_2024_1387_MOESM3_ESM.docx]

**Additional file 3 Primers for quantitative real-time PCR**

| Target gene | Primer Sequence (5’→3’) | Amplicon (bp) | source |
| --- | --- | --- | --- |
| tyrosine phosphatase (wzb) | TCGGTAATATATGCCGCTCCC | 109 | This study |
|  | CCAACTAAATGGCTACGCTCAG |  |  |
| UDP-N-acetyl-D-mannosamine dehydrogenase (wecC) | TAACGTATCCGATCTGCGAG | 179 | This study |
|  | AGAACACGACAACATCCGCT |  |  |
| transcriptional regulator, Phage repressor protein C (prpC) | TCCCATCAGCCTTTACCCCT | 152 | This study |
|  | TCATCTAATTCGATCCCCCA |  |  |
| alpha/beta hydrolase (hyd) | CATTTTACCTCTTACGCCCT | 149 | This study |
|  | TCGCGATCTGAAGTGCCTAC |  |  |
| Virulence associated trimeric autotransporter (vtaA31) | CGCTGAAGTAGTCGGACATGA | 144 | This study |
|  | GCACCACTATTTACCTCGGC |  |  |
| polysaccharide export protein (wza) | CGGTGAAAGCAAGTGATAACAT | 154 | This study |
|  | TTACCTGATTCAGCGGCAGA |  |  |
| 16s rRNA | CGGGAAACTGTCGCTAAT | 160 | Zhang et al. [29] |
|  | TGTGGCTGGTCATCCTCT |  |  |
