# Supplementary material for: Transcriptome analysis reveals a new virulence-associated trimeric autotransporter responsible for Glaesserella parasuis autoagglutination
Source: Vet Res. 2024 Oct 7;55:130. doi: 10.1186/s13567-024-01387-7 (PMC11460128; doi:10.1186/s13567-024-01387-7)
Supplement: Supplementary file 7 — Additional file 7: Amplification results of two “pathotyping” PCR assays. Information of amplification results of all virulence genes are listed. [file 13567_2024_1387_MOESM7_ESM.docx]

**Additional file 7 Amplification results of two “pathotyping” PCR assay**

| Virulence genes | Strains | | | | References |
| --- | --- | --- | --- | --- | --- |
|  | ZJ1208 | Δwza | C-Δwza | Δwza-vta |  |
| HPS_sp-sp | + | + | + | + | Howell et al. [36] |
| HPS_22976 | - | - | - | - |  |
| HPS_23060 | + | + | + | + |  |
| HPS_23879 | - | - | - | - |  |
| HPS_22970 | + | + | + | + |  |
| HPS_23505 | - | - | - | - |  |
| HPS_23300 | - | - | - | - |  |
| HPS_21059 | - | - | - | - |  |
| HPS_21058 | - | - | - | - |  |
| HPS_21068 | - | - | - | - |  |
| HPS_23887 | - | - | - | - |  |
| vtaAs (V) | + | + | + | + | Galofré-Milà et al. [37] |
| vtaAs (NV) | - | - | - | - |  |

(V): virulent strain amplified by primers of AV1-F+V1-R; (NV): non-virulent strain amplified by primers of AV1-F+NV1-R. “+”, present of the gene; “-”, absent of the gene.
